# Coronary artery involvement in a patient with IgG4-related disease

**DOI:** 10.1016/j.radcr.2023.07.062

**Published:** 2023-08-10

**Authors:** Ali Mohammadzadeh, Golnaz Houshmand, Hamidreza Pouraliakbar, Zeinab Soltani, Ghazaleh Salehabadi, Amir Azimi, Reza Shabanian

**Affiliations:** a Rajaie Cardiovascular Medical and Research Center, Iran University of medical sciences, Tehran, Iran; b Children`s Medical Center, Tehran University of Medical Sciences, Tehran, Iran

**Keywords:** Case report, IgG4-RD, Coronary artery, Ischemic cardiomyopathy, Computed tomography angiography, Cardiac magnetic resonance imaging

## Abstract

Immunoglobulin G4-related disease (IgG4-RD) is a chronic fibro-inflammatory disorder of obscure etiology characterized by significant infiltration of IgG4-positive plasma cells toward several organs. Coronary artery involvement is rarely seen in IgG4-RD patients; thereby, we aim to outline the noninvasive imaging findings of this rare case. Cardiac magnetic resonance (CMR) and coronary computed tomography angiography (CCTA) from a 15-year-old female diagnosed with IgG4-RD via histopathological assessment of orbital biopsy, were analyzed. CMR showed a severely reduced left ventricular ejection fraction and akinesia of the basal to mid-lateral, anterior, and septal walls. Inflammation of the basal to apical lateral wall and subendocardial infarction of the basal to apical lateral and mid inferoseptal walls were also evident. CCTA findings showed stenosis in branches of the left main artery (LM), left anterior descending artery (LAD), and right coronary artery (RCA), aortitis, and aortic wall thickening. After courses of proper treatment with prednisolone, Cellcept, and adalimumab, follow-up CMR showed significant improvement in LV systolic function and resolution of inflammation. Although IgG4-RD is an uncommon cause of coronary artery disease, it can cause lethal complications such as myocardial infarction. Hence, clinicians should be aware of cardiac complications in these patients.

## Introduction

Immunoglobulin G4-related disease (IgG4-RD) is an increasingly recognized chronic fibro-inflammatory disorder of unknown etiology that includes a set of conditions that have specific clinical, serological, and pathological features that can affect multiple organ systems and are characterized by the infiltration of IgG4-positive plasma cells, storiform fibrosis, and raised serum concentrations of IgG4 in the majority of cases. Its common manifestations include autoimmune pancreatitis (AIP), sclerosing cholangitis, sialadenitis, orbital region disease, retroperitoneal fibrosis, and chronic periaortitis. It most commonly affects middle-aged and older adults; however, it has also been reported in women and children [1], [2], [3].

Cardiovascular system involvement is less common in these patients and is often revealed as an incidental finding on extensive diagnostic imaging [3]. In cases of involvement, it tends to affect medium- to large-sized arteries. It rarely affects small arteries, like the coronary arteries. Coronary artery involvement can manifest as obstructive intraductal lesions, pseudotumors, or lymphoplasmacytic arteritis, predisposing to lethal complications like acute coronary syndrome (ACS) or sudden cardiac death. [2,4] Histopathological assessment is deemed the gold standard to confirm this condition's diagnosis. Nevertheless, when vital structures are involved, performing biopsies or obtaining surgical specimens can be challenging. Therefore, noninvasive coronary imaging evaluation with coronary CT angiography and non-invasive myocardial tissue characterization with cardiac MRI plays an essential role in diagnosing and managing the cardiac manifestation of the disease [2].

Since coronary artery involvement is rarely seen in IgG4-RD patients, we aim to outline the noninvasive imaging findings of this rare case.

## Case description

A 15-year-old Iranian girl diagnosed with an IgG4-related disease (IgG4RD) presented with dyspepsia, chronic fatigue, activity intolerance, and poor left ventricular (LV) function observed in echocardiography. Five years before referral, the patient had a history of left eye swelling, erythema, and proptosis, for which she underwent an orbital biopsy. The biopsy results revealed significant fibrosis and collagenization, along with the presence of chronic inflammatory cells. Immunohistochemistry (IHC) analysis demonstrated Cd138 positivity in background plasma cells, and IgG4 positivity in over 40% of plasma cells. The findings confirmed the diagnosis of chronic sclerosing IgG4-RD in the patient. Given the cardiac manifestations, she was referred to Rajaie Heart Center (RHC) for a comprehensive imaging assessment.

**Fig. 1:**
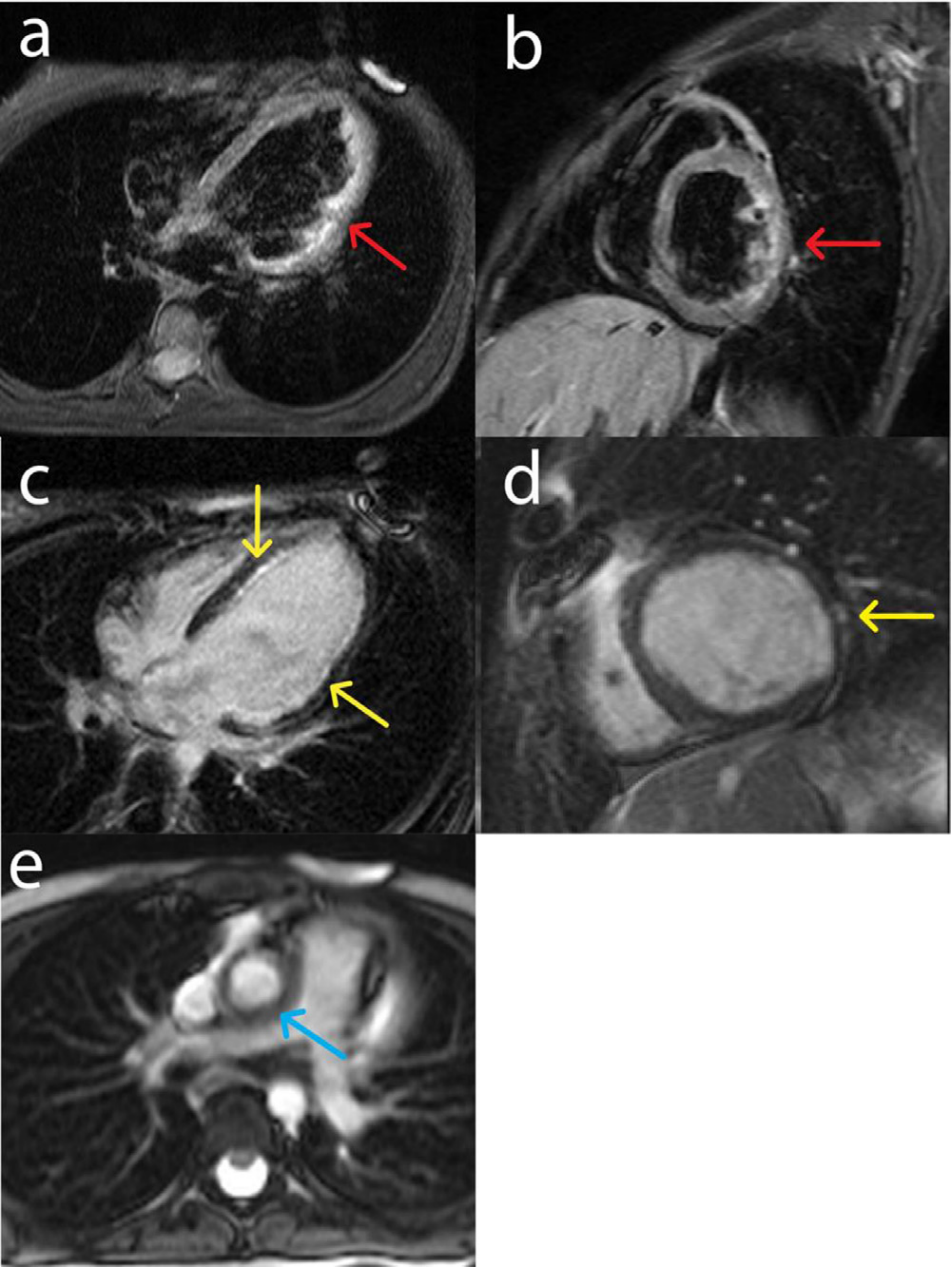
Representative images of CMR on admission before proper treatment. (a) STIR T2 increased signal intensity in the basal to apical lateral wall (red arrow) in 4 chambers view, (b) STIR T2 increased signal intensity in the basal to apical lateral wall (red arrow) in short axis view, (c) subendocardial enhancement in the basal to the apical lateral and mid inferoseptal wall (yellow arrow) in 4 chambers view, (d) subendocardial enhancement in the basal to the apical lateral and mid inferoseptal wall (yellow arrow) in short axis view (e) thickening of the aortic wall (blue arrow) in axial view.

**Fig. 2:**
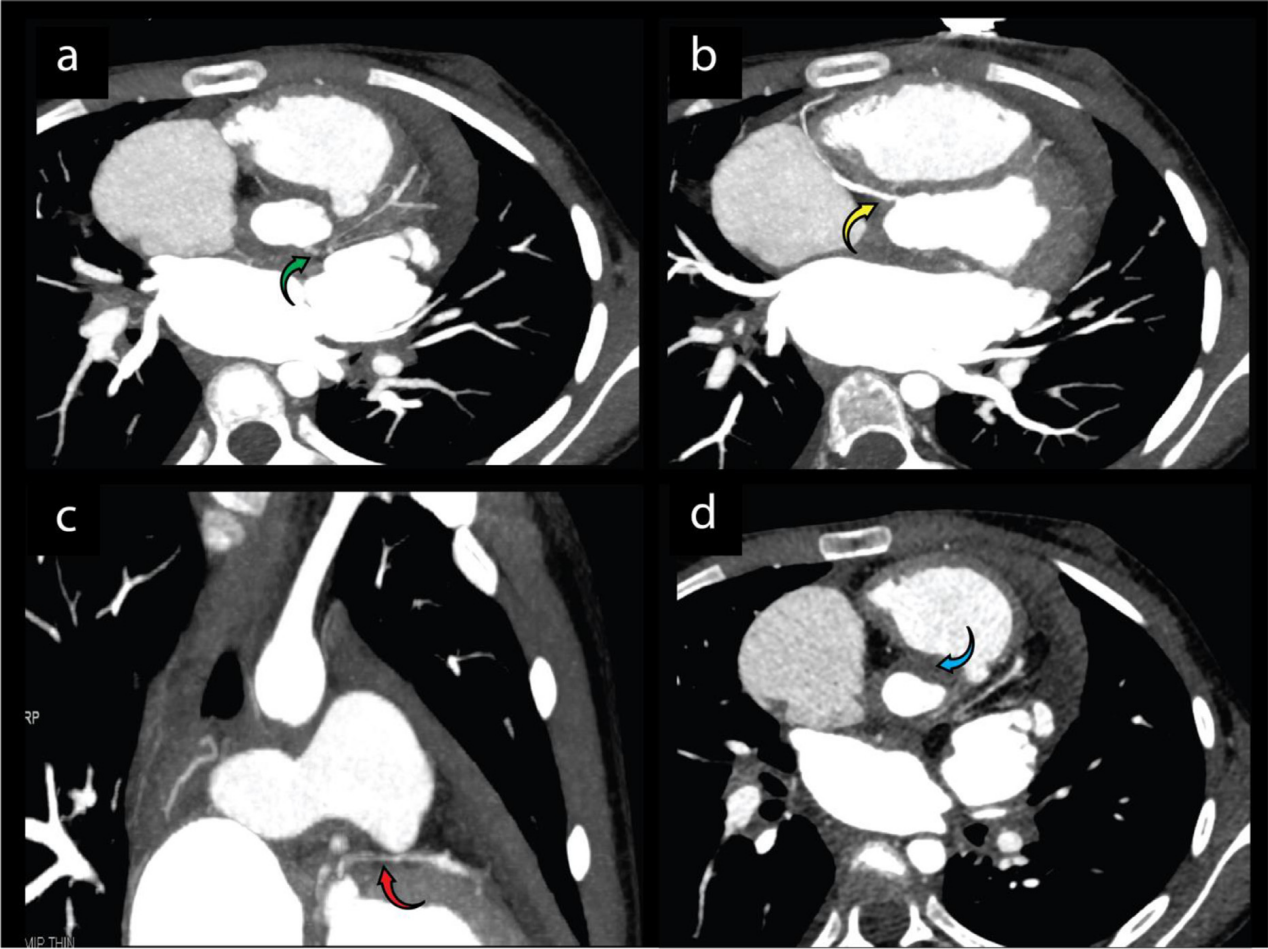
Representative images of CCTA findings before proper treatment. (a) Distal LM stenosis (green arrow) in axial view, (b) proximal RCA stenosis (yellow arrow) in axial view, (c) proximal LAD stenosis (red arrow) in coronal view, (d) circumferential thickening of the aorta (blue arrow) in axial view.

Cardiac magnetic resonance (CMR) showed a severely reduced LV ejection fraction (LVEF: 27%) and akinesia of the basal to apical lateral, mid to apical anterior, and septal walls. Increased signal intensity in T2-short tau inversion recovery (T2-STIR) imaging in the basal to apical lateral wall and subendocardial gadolinium enhancement (<50% of mural thickness) in the basal to apical lateral and mid-inferoseptal walls (Fig. 1). Considering the ischemic myocardial injury in CMR, coronary computed tomography angiography (CCTA) was performed. The findings showed severe ostial left main artery (LM) stenosis, long segmental left anterior descending artery (LAD) stenosis, right coronary artery (RCA) ostial stenosis, aortitis, and aortic wall thickening (7 mm) (Fig. 2).

**Fig. 3:**
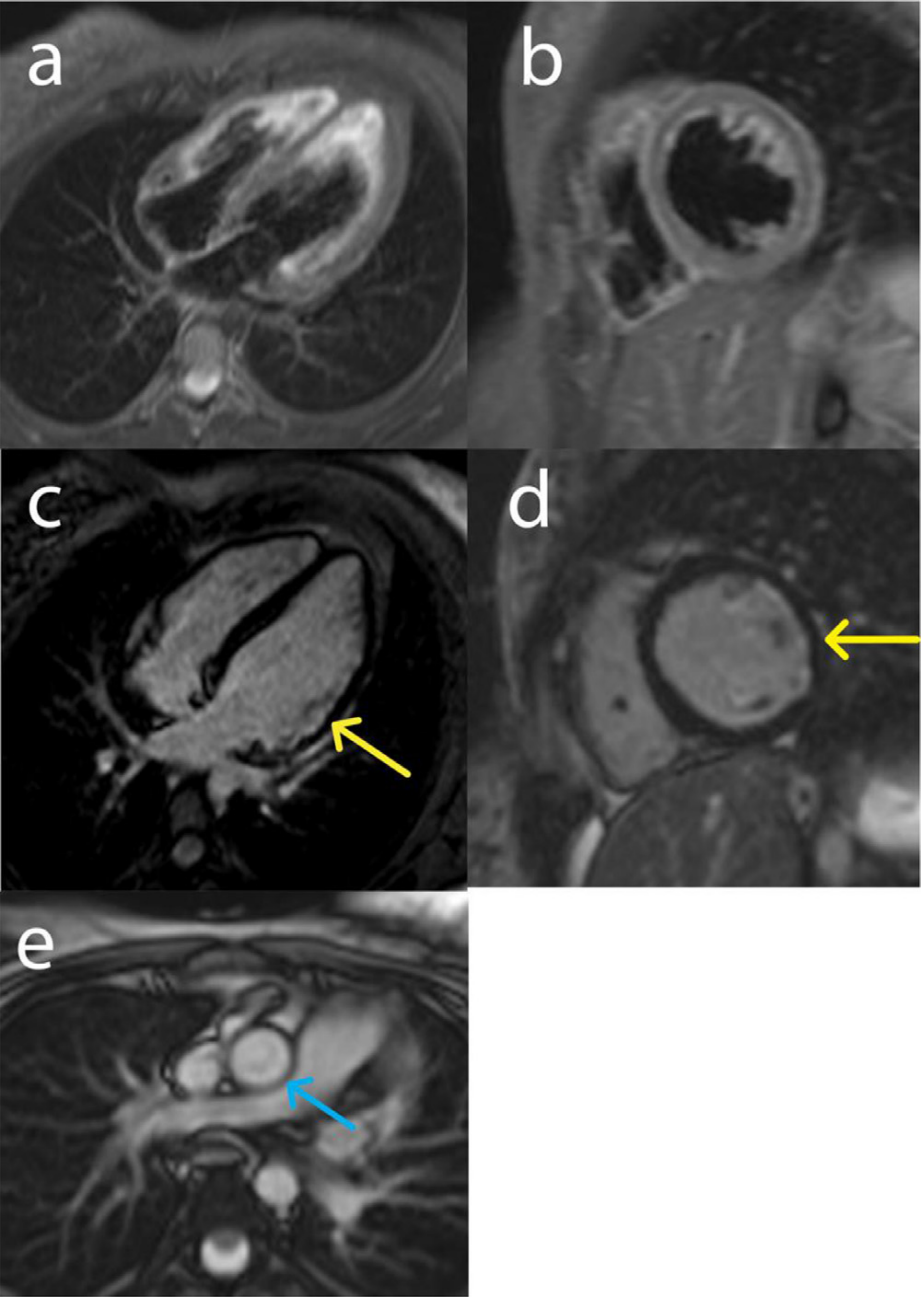
Representative images of the follow-up CMR after proper treatment. (a, b) STIR T2 no edema detected, (c, d) reduced subendocardial late gadolinium enhancement in the basal to the apical anterolateral and inferolateral wall (yellow arrows), (e) aorta with normal thickness after proper therapy (blue arrow).

The treatment began with 100 mg of aspirin daily, 5 mg of rosuvastatin daily, 0.625 mg of bisoprolol daily, 25 mg of prednisolone daily, 1500 mg of Cellcept daily, and 40 mg of adalimumab every 2 weeks. Upon discharge, the patient has been closely followed up to the present time. The follow-up CMR after 2 years of treatment showed significant improvement in LV function (LVEF: 37%) and resolution of inflammation; however, there was evidence of chronic subendocardial infarction in the basal to apical lateral walls. The thickness of the ascending aorta wall was also reduced (Fig. 3). The treatment not only improved cardiac functions but also alleviated the patient's clinical symptoms of the patient. Early diagnosis of IgG4-RD cardiac manifestations and proper treatment led to significant improvements in LV systolic function, edema, and periaortal wall thickness.

## Discussion

IgG4-RD is an immune-mediated systemic inflammatory condition characterized by significant infiltration of IgG4-positive plasma cells toward several organs. The concept of IgG4-RD was introduced around 2003 for the first time. Numerous conditions that were long considered to be unique entities are now thought to be included in the IgG4-RD spectrum, such as autoimmune pancreatitis, Riedel thyroiditis, Mikulicz's disease, and some types of tubulointerstitial nephritis [2,[5], [6], [7].

Aortitis or pericardial involvement is the most common cardiovascular presentation. In contrast, coronary artery involvement is rarely identified and difficult to diagnose, which includes various types such as obstructive lesions, periarterial thickening, aneurysms, dissections, and pseudotumor formation that can be completely asymptomatic or lead to lead to myocardial ischemia, aneurysmal rupture, heart blocks, and sudden cardiac death [2,[4], [5], [6], [7], [8]. In our case, stenosis was detected in the Ostial branches of LM and RCA and the long segmental branch of LAD.

Unlike rheumatologic diseases like rheumatoid arthritis and lupus in which systemic inflammation accelerates atherosclerosis and subsequently stenosis in the coronary artery, IgG4-RD coronary artery stenosis is caused by infiltration of IgG4 positive plasma cells by a nonatherosclerotic mechanism [8]. Overexpression of anti-inflammatory cytokines such as transforming growth factor-β (TGF-β) and interleukin ten and T-helper type-2-related cytokines like interleukins 4, 5, and 13 could be associated with the formation of the fibrotic arterial lesions in IgG4-related immune reactions [7,10].

The underlying pathology of coronary artery disease in IgG4-related disease involves excessive infiltration of IgG4-positive plasma cells, leading to fibrosis and thickening of the coronary arteries. On the other hand, atherosclerosis is characterized by the accumulation of plaque within the arterial walls. Distinguishing between IgG4-related disease and atherosclerosis using imaging can be challenging, but certain imaging features may help differentiate the 2 conditions. In IgG4-related disease, coronary CT angiography often reveals diffuse or segmental thickening of the coronary artery walls, affecting the entire circumference. Conversely, CT angiography in atherosclerosis typically demonstrates focal plaques within the coronary arteries, resulting in stenosis or narrowing of the vessel lumen. Coronary artery disease due to IgG4-RD is an underreported manifestation due to clinical misattribution to atherosclerotic coronary artery disease, especially in middle age men. This misdiagnosis could lead to an attempt at coronary revascularization, which can cause tissue dehiscence and anastomosis failure in the setting of percutaneous coronary intervention (PCI) and coronary artery bypass surgery (CABG) whether the underlying inflammatory condition is not controlled [4].

IgG4-RD diagnosis is complex and requires specific clinical, serological, radiological, and pathological findings defined as the comprehensive diagnostic criteria by Umehara et al. [11]. Serological findings demonstrate raised serum IgG4 concentration. Regardless, approximately 30% of patients demonstrate normal or mildly raised IgG4 serum levels, and this elevation is more common in patients with pancreatic disorder. Besides, elevated igG4 concentration is not specific for IgG4-RD and is also associated with other diseases like ANCA-associated vasculitis, lymphoma, pancreatic adenocarcinoma, and asthma. The histopathological finding also shows considerable infiltration and fibrosis of lymphocytes and plasmacyte and infiltration of IgG4-positive plasma cells (ratio of IgG4-positive cells to plasma cells >40 %, and >10 IgG4-positive plasma cells per high power field (HPF)). A histopathological investigation is deemed the gold standard for diagnosing IgG4-RD; nevertheless, it is not required for diagnosis confirmation as long as there is a strong suspicion from other findings due to difficult access to the sampled organ for biopsy [2,9]. In our case, a biopsy was taken from the left orbital mass and reported marked fibrosis and collagenization admixed with some chronic inflammatory cells. The surgical pathology report also demonstrates IgG4-positive in more than 40% of plasma cells and shows 10-12 IgG4-positive plasma cells /HPF that the histopathology was compatible with the chronic sclerosing IgG4-RD.

Preliminary diagnosis of coronary artery involvement can be obtained by conventional methods such as computed tomographic angiography (CTA) or invasive coronary angiography. Coronary CTA provides detailed visualization of the coronary arteries and displays the spectrum of IgG4-related coronary artery disease (CAD) such as stenosis, soft tissue formation, tumor-like lesion, coronary ectasia, and aneurysmal formation. In the context of IgG4-related CVD, CTA is particularly useful in identifying diffuse or partial arterial wall thickening (>2 mm) and homogeneous wall enhancement [1,^2^,^4^,^7^,^8^,^12^,13]. The present case showed no aneurysmal formations or tumor-like lesions, but stenosis and periarterial wall thickening.

CMR is a noninvasive imaging modality used to comprehensively assess cardiac structure and function in patients with IgG4-RD. It also plays a crucial role in evaluating disease activity and guiding treatment follow-ups. In a study conducted by Ratwatte et al., a series of IgG4-RD cases with cardiac involvement were examined using CMR. The CMR findings revealed several characteristic features associated with IgG4-RD, including myocardial thickening, patchy or diffuse enhancement (indicating the presence of fibrotic changes), and pericardial thickening or effusion. Furthermore, CMR has the ability to detect and characterize cardiac tumors, which can be an important manifestation of IgG4-RD [1,14]. In our present case, CMR shows evidence of severe LV systolic dysfunction and akinesia of the septal, and lateral walls with infarction in the septal and lateral wall mirroring the fibrotic lesions that are an essential indicator of ischemic myocardial injury and dysfunction.

Glucocorticoids are considered first-line-therapy that is also used for remission maintenance though the majority of patients experience disease relapse during or after glucocorticoid tapers. Therefore, B cell-depleting therapy such as rituximab or adalimumab may also be required [2], [3], [4].

## Conclusion

IgG4-RD is an uncommon cause of coronary artery disease but can lead to life-threatening complications including myocardial infarction. Regarding the importance of early treatment, clinicians should be aware of cardiac complications in these patients. Besides, IgG4-RD should be a concern in young patients in cases of myocardial ischemia.

## Patient consent

The written informed consent for publication of this case report has been obtained from the patient. The agreement document will remain with us for our own records.
